# Amino Acid Composition, Molecular Weight Distribution and Gel Electrophoresis of Walnut (*Juglans regia* L.) Proteins and Protein Fractionations

**DOI:** 10.3390/ijms15022003

**Published:** 2014-01-27

**Authors:** Xiaoying Mao, Yufei Hua, Guogang Chen

**Affiliations:** 1 Food College, Shihezi University, Shihezi 832003, Xinjiang, China; E-Mail: maoxiaoying99@163.com; 2 State Key Laboratory of Food Science and Technology, School of Food Science and Technology, Jiangnan University, Wuxi 214122, Jiangsu, China; E-Mail: yfeihua@163.com

**Keywords:** walnut proteins, fractionation, amino acid composition, molecular weight distribution, 2-DE, HPLC

## Abstract

As a by-product of oil production, walnut proteins are considered as an additional source of plant protein for human food. To make full use of the protein resource, a comprehensive understanding of composition and characteristics of walnut proteins are required. Walnut proteins have been fractionated and characterized in this study. Amino acid composition, molecular weight distribution and gel electrophoresis of walnut proteins and protein fractionations were analyzed. The proteins were sequentially separated into four fractions according to their solubility. Glutelin was the main component of the protein extract. The content of glutelin, albumin, globulin and prolamin was about 72.06%, 7.54%, 15.67% and 4.73% respectively. Glutelin, albumin and globulin have a balanced content of essential amino acids, except for methionine, with respect to the FAO pattern recommended for adults. SDS-PAGE patterns of albumin, globulin and glutelin showed several polypeptides with molecular weights 14.4 to 66.2 kDa. The pattern of walnut proteins in two-dimension electrophoresis (2-DE) showed that the isoelectric point was mainly in the range of 4.8–6.8. The results of size exclusion chromatogram indicated molecular weight of the major components of walnut proteins were between 3.54 and 81.76 kDa.

## 1. Introduction

Walnut (*Juglans regia* L.) is the most widespread tree nut in the world [1]. China is the largest producer of walnut in the world. According to the Food and Agriculture Organization (FAO), UN 2013 statistics [2], Chinese walnut production reached 1655.508 thousand tons in 2011, accounting for 48.42% of the world’s total production of walnut, ranking first in the world production of walnut. In China, walnuts (*Juglans regia* L.) are a resourceful nut and distributed widely. Walnuts, the seeds of *Juglans regia* L., are a highly nutritious food. It has been reported frequently that regular consumption of walnuts can decrease the risk of heart disease [3]. The FDA authorized a health claim indicating that diets including walnuts can reduce the risk of heart disease [4]. As a kind of health food, walnuts are often utilized as ingredient of many foodstuffs such as bakery products to enhance the nutrition value [5]. In the past years studies have shown that the content of oil in walnut is up to 62%~68%, which contains a high content of monounsaturated and polyunsaturated fatty acids [6]. Anderson *et al.* reported that besides walnut oil, some other unknown components of walnuts are also helpful to health [7]. Besides oil, walnuts contains 24% protein, 12%–16% carbohydrates, 1.5%–2.0% cellulose, and 1.7%–2.0% mineral [8–12]. Some studies of walnut proteins have been conducted [10,11,13–15], however, little information exists on the detail of the properties of walnut proteins. The composition and characteristics of walnut proteins is the key to further understanding of the nature of walnut proteins and protein isolates, which can better solve application problems regarding walnut proteins.

This research team also studied the composition and characteristics of safflower (*Carthamus tinctorius*) seed proteins. As a kind of health food, the chemical composition and protein characteristics of walnuts should also be investigated deeply. To make full use of the protein resource, a comprehensive understanding of compositions and characteristic of walnut proteins is required. The objectives of the present work were to determine amino acid composition, molecular weight distribution and gel electrophoresis of walnut (*Juglans regia* L.) proteins and protein fractionations.

## 2. Results and Discussion

### 2.1. Chemical Compositions

The proximate compositions of walnut kernel and defatted walnut flour were shown in Table 1. Protein content of walnut kernel was higher than of that grown in USA (16.66%) (*p* < 0.05) reported by Sze-Tao [11], with value of 17.66%. The different protein content may be responsible for the region in which walnut was planted. Walnut kernel contained 60.84% fat while defatted walnut flour contained 1.80%. It was indicated that the defatting procedure could reduce fat content of samples effectively. Defatted walnut flour was used as starting material for the preparation of walnut protein isolates. The Nitrogen Solubility Index (NSI) of defatted walnut flour was 7.64%, which was lower than that of peanut protein isolate (60.5%), or soy protein isolate (71.7%) as reported by Yu *et al.* [16], and Molina *et al.* [17], respectively. It was mainly because gluten protein is major component of walnut proteins.

### 2.2. Protein Fractionation

**Figure 1.**
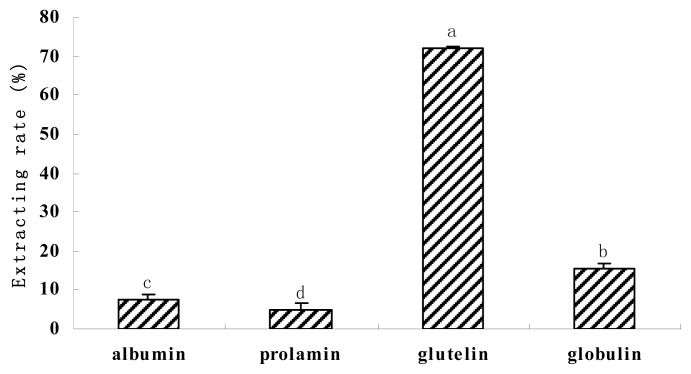
Contents of walnut proteins fractions. Bars indicate the standard deviation from triplicate determinations. Different letters indicate significant differences (*p* < 0.05).

The classification of seed proteins according to their solubility was developed by Osborne [18], who distinguished four different fractions: albumin (water-soluble), globulin (salt-soluble), prolamins (alcohol-soluble) and glutelins (soluble in dilute NaOH). The proportion of walnut proteins fractions is shown in Figure 1. Glutelin was found to be the major protein fraction of walnut kernel, which was much more than that reported by Sze-Tao [11]. The different walnut variety studied may be responsible for the unagreements. The content of glutelin, albumin, globulin and prolamin were 72.06%, 7.54%, 15.67% and 4.73% of the total extractable protein, respectively.

### 2.3. Amino Acid Compositions

Amino acid composition is an important chemical property of proteins, as it determines their nutritional value. The amino acid compositions of walnut proteins were measured and the results are shown in Table 2. The walnut proteins contain a relatively low content of lysine and high levels of arginine. This agreed with the study of Ruggeri [19]. In addition, the amino acid pattern of Chinese walnuts reported in this work differed form the values of the other cultivars in Italy [19] and in New Zealand [20]. Glutelin, albumin, globulin and defatted walnut flour had a balanced content of essential amino acids, except for methionine, with respect to the FAO recommended pattern for adult [21]. From these results we concluded that walnut proteins could be a good source of essential amino acids for adults.

### 2.4. Gel Electrophoresis

**Figure 2.**
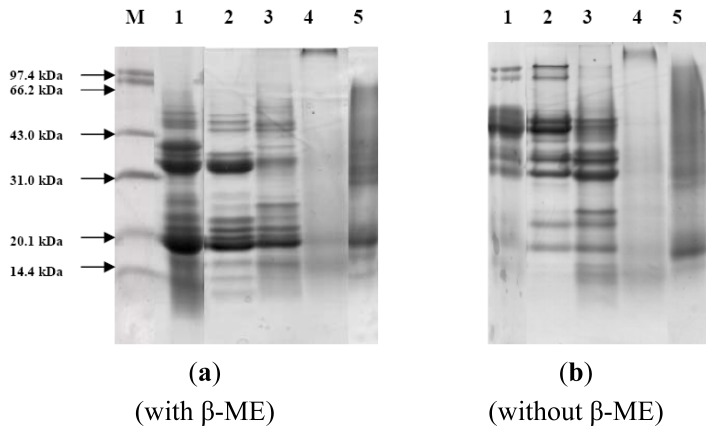
The SDS-PAGE profiles of walnut proteins and four protein fractions. (**a**) Proteins extracted under reducing condition (SDS + β-mercaptoethanol); (**b**) Proteins extracted under dissociating condition (SDS). M: low molecular weight markers; 1, defatted walnut flour; 2, globulin; 3, albumin; 4, prolamin; 5, glutelin. Protein samples were loaded at 30 μg on each lane and proteins were detected with standard Coomassie blue stain after electrophoresis.

In order to characterize the proteins, presence of proteins’ number of bands, approximate molecular weights, and the sub-unit patterns were studied by means of SDS-PAGE. SDS-PAGE was performed in the presence and absence of a reducing agent, β-mercaptoethanol (β-ME). This also allowed us to distinguish between free polypeptide chains and the chains linked by disulphide bridges. The SDS-PAGE profiles of walnut proteins and four protein fractions in the presence and absence of β-ME were presented in the reducing condition (Figure 2a) and non-reducing condition (Figure 2b). The results showed that walnut proteins contained nine bands in non-reducing condition (lane 1, Figure 2b). Compared with the results in the non-reducing condition, walnut proteins showed at least eleven bands obviously and three bands were stained strongly with the molecular weights of 20, 31, and 40 kDa (lane 2, Figure 2a). Many weakly stained bands were clear with the molecular weight range from 14.4 to 66.2 kDa. A major band with molecular weight of 40~60 kDa was stained weakly in reducing condition. This result indicated these subunits are formed by disulfide bond-linked polypeptides with molecular masses 40–45, 20–25 and 25–30 kDa, respectively. And also, a major band consisting of a broad zone, with molecular weights of 31–43 kDa appeared in both reducing and non-reducing conditions. This indicated that these major polypeptides were free of inter-chain disulphide bonds. Albumin showed eight bands with the major bands distributed at the molecular weights of 20.1 kDa (lane 4, Figure 2a). Globulin showed nine bands with the two major bands at the molecular weights of 20.1 and 35.0 kDa (lane 3, Figure 2a). The results of albumin and globulin were congruent with a previous report of Sze-Tao [11]. Glutelin showed five bands obviously with the major bands at the molecular weights of 20.1 and 30 kDa (lane 6, Figure 2a). Interestingly, prolamin did not show any bands (lane 5, Figure 2a) whatever the reducing condition and non-reducing condition. This was also congruent with the reports of Sze-Tao [11]. Albumin, globulin and glutelin showed many bands concentrated at the molecular weight range from 14.4 to 66.2 kDa.

### 2.5. 2-DE

**Figure 3.**
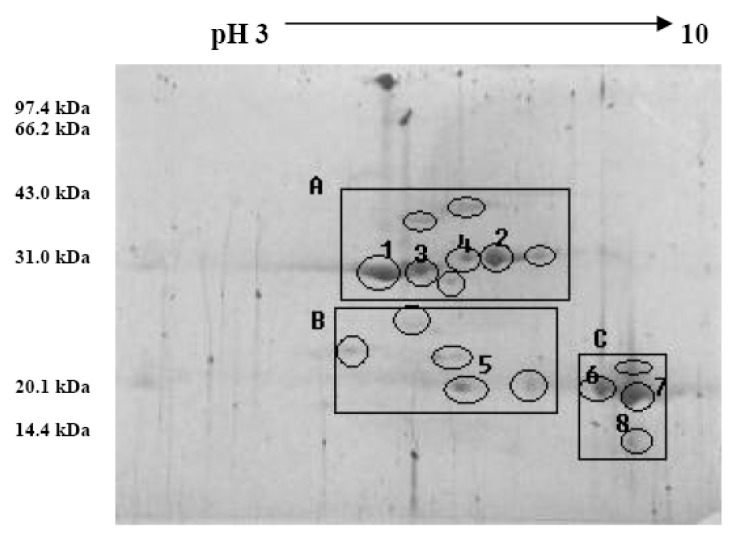
Two-dimensional gel electrophoresis of walnut proteins. Separation was performed in the pH region of 3–10 and followed by staining using blue colloidal method. Major proteins appeared were encoded.

The pattern of walnut proteins in 2-DE analysis is presented in Figure 3; it exhibited clear walnut proteins profiles and displayed less contamination in 2-DE gels. It also exhibited better separation for middle-*M*_r_ proteins (approximately 30.0–43.0 kDa; region A) and detected more protein spots with intensity for low-*M*_r_ proteins region (approximately 14.4–20.1 kDa; region C). The results show that, the isoelectric points of the walnut proteins were mainly concentrated between the pI 4.8–6.8, which was acidic protein; and a few of the proteins are basic proteins, in which pI values were mainly concentrated from 8.4 to 9.0.

### 2.6. High Performance Size Exclusion Chromatography

**Figure 4.**
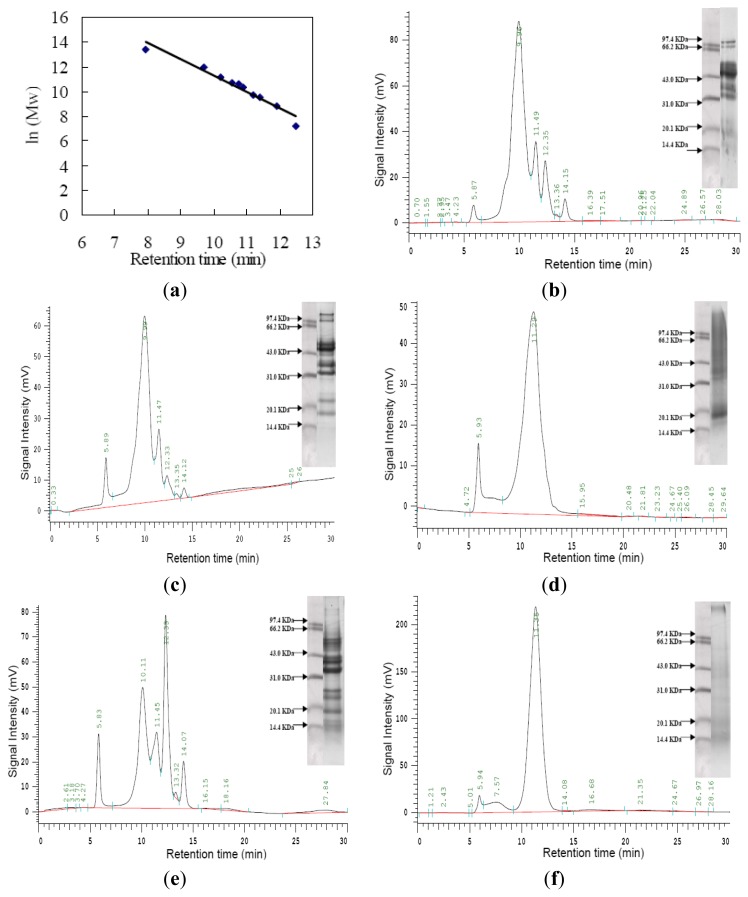
Molecular weight distribution of defatted walnut flour and walnut proteins fractions. (**a**) The calibration curve of standard proteins; (**b**) Defatted walnut flour; (**c**) Globulin; (**d**) Glutelin; (**e**) Albumin; (**f**) Prolamin. Figure inside was SDS-PAGE without β-mercaptoethanol. A calibration curve of 10 standard proteins was used for interpreting the results. Ten standard proteins were thyroglobulin (*M*_W_: 669, 000), aldolase (*M*_W_: 158,000), BSA (*M*_W_: 67,000), ovalbumin (*M*_W_: 43,000), peroxidase (*M*_W_: 40,200), adenylate kinase (*M*_W_: 32,000), myoglobin (*M*_W_: 17,000), ribonuclease A (*M*_W_: 13,700), aprotinin (*M*_W_: 6500), and vitamin B12 (*M*_W_: 1350), respectively.

The size exclusion chromatogram using a high-performance liquid chromatogram system was used to study molecular weight distribution of walnut proteins and the results are shown in Figure 4a–f. Molecular weight was estimated from the calibration curve of standard protein for the column. The five peaks of walnut proteins were characterized by retention time around 5.87, 9.96, 11.49, 12.35, and 14.15 min, respectively, corresponding to the *M*_W_ (content) of 17,627.91 kDa (2.03%), 81.76 kDa (70.84%), 10.95 kDa (12.83%), 3.54 kDa (10.36%) and 0.33 kDa (3.31%) (Figure 4b). While, HPLC profiles of globulin (Figure 4c), glutelin (Figure 4d) and albumin (Figure 4e) showed a small peak with the retention time of 5.8min, suggesting that the observed peak of higher molecular weight (16,291.69 kDa) was probably the aggregations of walnut proteins. Disulfide bonds between the protein molecules may be due to aggregate forms [22]. Globulin showed five peaks, corresponding to the *M*_W_ of 17,170.75 kDa (7.81%), 78.60 kDa (70.20%), 11.25 kDa (12.37%), 3.63 kDa (4.15%) and 0.35 kDa (1.06%), respectively (Figure 4c). Besides the peak of higher molecular weight (16,291.69 kDa), glutelin showed another big peak, corresponding to the molecular weight of 14.25 kDa respectively, and its relative intensity was of 87.30% (Figure 4d). Moreover, albumin showed five major peaks, and corresponded to the molecular weight (content) of 18,579.07 kDa (7.61%), 67.14 kDa (34.73%), 11.54 kDa (16.58%), 3.63 kDa (30.54%) and 0.37 kDa (5.88%), respectively (Figure 4e). Prolamin (Figure 4f) showed three peaks, corresponding to the molecular weight of 16,079.05, 1888.94 and 13.17 kDa, with the relative intensity of 2.59%, 6.84%, and 88.09%, respectively. The results of a size exclusion chromatogram indicated that molecular weight of walnut proteins were wide in range, with the major composition of MW between 3.54 and 81.76 kDa. The generation of a small amount of protein aggregates may also due to the low Nitrogen Solubility Index (NSI) of defatted walnut flour.

## 3. Experimental Section

### 3.1. Materials and Methods

Walnuts (*Juglans regia* L.) were purchased from Xinjiang in China. The defatted walnut flour was produced according to the method suggested by Sze-Tao and Sathe [11] for the extraction of walnut proteins. Walnut was ground in a Waring Blender. The flour was defatted with hexane (flour/hexane ratio of 1:10 *w/v*) under constant magnetic stirring for 3 h. The slurry was vacuum filtered through filter paper and the residue was used for subsequent extraction. Hexane extractions were repeated until the filtrate was clear. Residues from the last extraction and filtration step were air dried in a fume hood. The defatted walnut flour was ground to 150 meshes with Waring Blender and stored at −20 °C for further use.

### 3.2. Proximate Analysis

Moisture, fat and ash contents were determined according to the methods of AOAC [23], numbers 950.46, 960.39, and 920.153, respectively. The protein contents of samples were determined by the micro-Kjeldhal method [24] through the use of the protein-nitrogen coefficient of 5.30 [11]. Carbohydrates were determined according to the method of Zhu *et al.* [25]. The contents were expressed on a dry weight basis. Each analysis was done in triplicate, and data were reported as means ± standard deviation.

### 3.3. Protein Fractionation

Protein was extracted sequentially with 1.0 M NaCI (albumin + globulin), then 70% ethanol (prolamin) and finally, 0.1 M NaOH (glutelin) (defatted flour/solvent ratio of 1:10 *w/v*) for 1 h at 25 °C under constant magnetic stirring. The slurry was then centrifuged (13,000× *g*, 4 °C, 15 min) and the supernatant was vacuum filtered using filter paper to remove insoluble particles. The fractions were then dialyzed against water and lyophilized. All lyophilized protein fractions were stored in airtight plastic bottles at −20 °C until further use. Extracting rate was calculated as: lyophilized protein fractions divided by total protein.

### 3.3. Amino Acid Composition

Amino acids analysis was determined according to the method of Zhu, Zhou and Qian [25]. Samples (0.1 g) were subjected to acid hydrolysis with 5 mL of 6 M HCl under nitrogen atmosphere for 24 h at 110 °C. Each hydrolysate was washed into a 50 mL volumetric flask and made up to the mark with distilled water. The amino acids were subjected to RP-HPLC analysis (Agilent 1100, Agilent Technologies Co. Ltd., Palo Alto, CA, USA) after precolumn derivatisation with *O*-phthaldialdehyde (OPA) or with 9-fluorenylmethyl chloroformate (FMOC). Methionine and cysteine were determined separately by oxidation products before hydrolysis in 6 M HCl. Amino acid compositions were reported as g of amino acid/100 g of protein.

### 3.4. Gel Electrophoresis

Protein subunits compositions were analyzed by sodium dodecyl sulphate polyacrylamide gel electrophoresis (SDS-PAGE, Beijing Liuyi Instrument Factory, Beijing, China). SDS-PAGE was performed according to the method of Laemmli [26] by the discontinuous buffer system at 4% stacking gel concentration and 12.5% separating gel concentration, using gel electrophoresis apparatus DYCZ-30 (Beijing Liuyi Instrument Factory, China). Electrophoresis was carried out in the presence and absence of β-mercaptoethanol (2% *v/v*) [27]. Samples were extracted with SDS-PAGE buffer for four hours at room temperature (RT) using a vortex mixing. After heating in boiling water bath for 10 min, Samples were cooled to RT and then centrifuged (10,000× *g*, 10 min, RT). Supernatants were used for electrophoresis. The electrophoresis was run at 15 mA in the stacking gel and 25 mA in the separating gel until the tracking dye reached the bottom of the gel and gels were stained with Coomassie Brilliant Blue G 250. Molecular weight of subunits were estimated by using a LMW calibration kit (Shanghai Institute of Biochemistry, Shanghai, China) consisting of hen egg white lysozyme (14.4 kDa), trypsin inhibitor (20.1 kDa), bovine carbonic anhydrase (31.0 kDa), rabbit actin (43.0 kDa), bovine serum albumin (66.2 kDa), and rabbit phosphorylase b (97.4 kDa).

### 3.5. 2-DE

2-DE was carried out using Protean IEF Cell (Bio-Rad, Hercules, CA, USA) for 1st dimension and Protean II xi Cell for 2nd dimension. First, the defatted walnut flour was defatted by Chloroform-methanol mixture (2:1, *v/v*). Then, protein powder sample was prepared based on the trichloroacetic acid (TCA)/acetone precipitation method described by Damerval *et al.* [28]. The powdered sample (1 g) was added in 10 ml precipitation solution (10% TCA and 0.07% β-ME in cold acetone), homogenized and sonicated in ice bath for 10 min, and then precipitated at −20 °C overnight. It was centrifuged at 15,000× *g* for 15 min at 4 °C. The precipitated proteins were washed with ice-cold acetone containing 0.07% β-ME to remove pigments and lipids until the supernatant was colorless. Then, repeated the previous operation. Pellets were vacuum-dried and then were resuspended in lysis buffer [8 mol L^−1^ urea, 4% (*w/v*) 3-[(3-Cholamidopropyl) dimethylammonio] propanesulfonic acid (CHAPS), 65 mmol L^−1^ dithiothreitol (DTT), and 0.2% Bio-Lyte, 0.001% bromophenol blue. After centrifugation at 12,000× *g* for 30 min, the supernatant was collected. Protein concentration was determined according to the Bradford method [29]. Polyacrylamide gel strips with an immobilized pH gradient of 3–10 (70 mm, GE Healthcare Bio-Sciences AB, Uppsala, Sweden, Cat.# 17-1233-01) were used for 1st dimension. The second dimensional electrophoresis (SDS-PAGE) was performed as described by Laemmli [26]. The focused gel was transferred onto 12% (*w/v*) SDS-PAGE self-cast gels. Electrophoresis was carried out at 20 mA per gel for 30 min and 15 mA per gel until the dye had reached the bottom of the gel. The gels were stained by the method of Candiano *et al.* [30].

### 3.6. Molecular Weight Distribution by SEC-HPLC

The molecular weight distribution was determined by High performance size exclusion chromatography (SEC-HPLC, Waters Chromatography Division, Milford, MA, USA). Four walnut proteins (5 mg/mL) were extracted by sodium phosphate buffer (0.05 M, pH 8.0) containing sodium chloride (0.3 M) for 4 h at 25 °C under constant magnetic stirring and then were centrifuged at 10,000× *g* for 10 min (25 °C). The supernatant was filtered through a cellulose acetate membrane with a pore size of 0.45 μm (Sartorius Co., Ltd., Gottingen, Germany). A Waters 2690 liquid chromatogram system (Waters Chromatography Division, Milford, MA, USA) equipped with a Shodex protein KW-804 column (Shodex Separation and HPLC Group, Tokyo, Japan) and a Waters 996 photodiode array detector were used to determine the molecular weight distribution. The flow rate was 1 mL/min using phosphate buffer (0.05 mol/L, 0.3 mol/L NaCl, pH 7.0) as the mobile phase. About 10 μL protein solutions were injected into the column and the eluent was monitored at 280 nm. All samples were measured in triplicate and the representative examples were selected for discussion. A calibration curve of 10 standard proteins was used for interpreting the results.

### 3.7. Statistical Analysis

All analyses were done in triplicate, and data were reported as means ± standard deviation. Where appropriate, data were analyzed for significance using analysis of variance and Fisher’s least significant difference (LSD at a 5% significance level) by General Linear Model of SPSS (Software version 11.0, SPSS, Chicago, IL, USA).

## 4. Conclusions

A classification method of seed proteins by Osborne to fractionation of walnut proteins, was proposed. Glutelin was found to be the major protein fraction of walnut kernel. Amino acids analysis results implied that walnut proteins could be a good source of essential amino acids for adults.

SDS-PAGE electrophoresis was used to confirm the number of bands, approximate molecular weights, and the sub-unit patterns of walnut proteins, glutelin, albumin, globulin and prolamin. This result indicated subunits of walnut proteins with molecular weights of 40–60 kDa were formed by disulfide bond-linked polypeptides with molecular masses 40–45, 20–25, and 25–30 kDa, respectively. And also, a major band of walnut proteins sample with molecular weights 31–43 kDa, were free of interchain disulphide bonds. Albumin, globulin and glutelin showed many bands concentrated at the molecular weight range from 14.4 to 66.2 kDa.

The pattern of walnut proteins by 2-DE analysis was investigated for the first time. The isoelectric point and acid-alkaline properties of walnut proteins have not been reported up to now. The results showed that the isoelectric point of the walnut proteins was mainly concentrated between the pI 4.8 and 6.8, which was acidic protein; a few of the proteins are basic proteins, which have pI values mainly concentrated from 8.4 to 9.0. Molecular weight distribution of walnut proteins and protein fractions were investigated by the size exclusion chromatogram analysis. The results indicated molecular weights of walnut proteins were wide in range, with the major composition of MW between 3.54 and 81.76 kDa.

As a kind of food protein, walnut proteins are complex, with a wide range of molecular weights and multiple subunits. It was suggested that protein separated from walnuts can be useful as important content of functional protein. The results reported here may represent relevant information about walnut proteins, which are considered to be used as an additive food ingredient (such as in cake, ice cream and desserts) or in the form of novel foods in the food processing industry.

## Figures and Tables

**Table 1.** Chemical compositions of walnut kernel and defatted walnut flour a.

Materials	Protein (%)	Fat (%)	Ash (%)	Moisture (%)	Carbohydrate (%)	NSI b (%)
Walnut kernel	17.66 ± 0.42	60.84 ± 1.04	1.94 ± 0.02	3.20 ± 0.01	16.36 ± 0.12	——
Defatted walnut flour	52.51 ± 0.33	1.80 ± 0.12	0.54 ± 0.02	9.20 ± 0.02	35.95 ± 0.16	7.64 ± 0.12

a Results represent the average of three determinations ± SD, values in the same column with different letters are significantly different (*p* < 0.05);

b NSI, Nitrogen solubility index.

**Table 2.** Amino acid composition of walnut proteins and protein fractions.

Amino acids	Walnut protein	Albumin	Globulin	Prolamin	Glutelin	*p*-values	FAO/WHO (1990)
Asp	10.04 ± 0.43 ^b^	8.02 ± 0.57 ^c^	7.13 ± 0.51 ^c^	18.08 ± 0.42 ^a^	10.51 ± 0.44 ^b^	<0.0001	
Glu	22.16 ± 0.4 ^c^	28.7 ± 3.36 ^b^	28.8 ± 1.26 ^b^	33.03 ± 1.06 ^a^	22.7 ± 2.05 ^c^	0.01126	
Ser	5.84 ± 0.12 ^a^	4.8 ± 0.36 ^b^	5.75 ± 0.23 ^a^	3.22 ± 0.12 ^c^	5.81 ± 0.20 ^a^	0.00197	
His	2.38 ± 0.26 ^a^	2.23 ± 0.14 ^a^	2.01 ± 0.05 ^a^	1.4 ± 0.35 ^b^	2.19 ± 0.16 ^a^	0.03921	1.9 (1.6)
Gly	5.43 ± 0.07 ^d^	5.89 ± 0.17 ^c^	8.73 ± 0.17 ^a^	7.68 ± 0.27 ^b^	5.28 ± 0.25 ^d^	0.0006	
Thr	3.58 ± 0.20 ^a^	2.64 ± 0.07 ^b^	2.02 ± 0.07 ^c^	1.59 ± 0.13 ^d^	3.49 ± 0.04 ^a^	<0.0001	3.4 (0.9)
Arg	14.73 ± 0.42 ^c^	15.67 ± 0.34 ^b^	16.01 ± 0.33 ^b^	17.52 ± 0.43 ^a^	13.47 ± 0.33 ^d^	0.00848	
Ala	4.74 ± 0.19 ^a^	3.29 ± 0.24 ^b^	2.62 ± 0.34 ^c^	2.57 ± 0.18 ^c^	4.73 ± 0.27 ^a^	0.00224	
Tyr	2.76 ± 0.11 ^b^	2.53 ± 0.06 ^c^	0.76 ± 0.07 ^d^	3.72 ± 0.09 ^a^	2.83 ± 0.09 ^b^	<0.0001	
Cys	0.84 ± 0.08 ^c^	2.21 ± 0.10 ^a^	1.97 ± 0.09 ^b^	2 ± 0.04 ^b^	0.56 ± 0.09 ^d^	<0.0001	
Val	4.18 ± 0.14 ^a^	3.24 ± 0.11 ^b^	3.05 ± 0.16 ^b^	1.49 ± 0.16 ^c^	4.15 ± 0.16 ^a^	0.00026	3.5 (1.3)
Met	1.16 ± 0.12 ^c^	1.7 ± 0.10 ^b^	2.32 ± 0.08 ^a^	0.84 ± 0.14 ^d^	1.55 ± 0.11 ^b^	0.00119	2.5 (1.7)
Phe	4.94 ± 0.23 ^a^	3.89 ± 0.15 ^b^	3.78 ± 0.08 ^b^	1.92 ± 0.10 ^d^	5.11 ± 0.1 ^a^	<0.0001	6.3 (1.9)
Ile	3.28 ± 0.15 ^a^	2.66 ± 0.16 ^b^	2.79 ± 0.13 ^b^	0.94 ± 0.07 ^c^	3.32 ± 0.17 ^a^	<0.0001	2.8 (1.3)
Leu	7.13 ± 0.12 ^a^	5.21 ± 0.11 ^b^	5.48 ± 0.16 ^b^	1.51 ± 0.13 ^c^	7.31 ± 0.26 ^a^	<0.0001	6.6 (1.9)
Lys	2.58 ± 0.12 ^b^	3.31 ± 0.16 ^a^	2.52 ± 0.16 ^b^	0.83 ± 0.10 ^d^	1.7 ± 0.17 ^c^	0.00139	5.8 (1.6)
Pro	4.22 ± 0.29 ^b^	4.03 ± 0.10 ^b^	4.27 ± 0.13 ^b^	1.64 ± 0.11 ^c^	5.3 ± 0.24 ^a^	<0.0001	

All amino acid (AA) values are expressed as grams per 100 g of protein; Numbers in parentheses of FAO/WHO recommended pattern (1990) represent essential amino acid for adults, and numbers outside the parentheses represent essential amino acid for pre-school child (2–5 years); Values are means ± SD of three determination. Different letters in the same row indicate significant differences (*p* < 0.05).
